# In vitro production of naked mole-rats’ blastocysts from non-breeding females using in vitro maturation and intracytoplasmic sperm injection

**DOI:** 10.1038/s41598-023-49661-6

**Published:** 2023-12-15

**Authors:** Raffaella Simone, Daniel Čižmár, Susanne Holtze, Geert Michel, Anje Sporbert, Charlotte Okolo, Thomas B. Hildebrandt

**Affiliations:** 1 Leibniz Institute for Zoo and Wildlife Research (IZW) in the Forschungsverbund Berlin eV, Reproduction Management, Alfred-Kowalke-Straße, Berlin, Germany; 2 Freie Universitaet Berlin, Veterinary Medicine, Berlin, Germany; 3 FEM, Transgenic Technologies, Charité–Universitätsmedizin, Berlin, Germany; 4 Advanced Light Microscopy Technology Platform (Max Delbrück Center for Molecular Medicine), Campus Buch, Berlin, Germany

**Keywords:** Embryogenesis, Embryology

## Abstract

The African naked mole-rat (*Heterocephalus glaber*) is an attractive model for cancer and aging research due to its peculiar biological traits, such as unusual long life span and resistance to cancer. The establishment of induced pluripotent stem cells (iPSCs) would be a useful tool for in vitro studies but, in this species, the reprogramming of somatic cells is problematic because of their stable epigenome. Therefore, an alternative approach is the derivation of embryonic stem cells from in vitro-produced embryos. In this study, immature oocytes, opportunistically retrieved from sexually inactive females, underwent first in vitro maturation (IVM) and then in vitro fertilization via piezo-intracytoplasmic sperm injection (ICSI). Injected oocytes were then cultivated with two different approaches: (i) in an in vitro culture and (ii) in an isolated mouse oviduct organ culture system. The second approach led to the development of blastocysts, which were fixed and stained for further analysis.

## Introduction

Naked mole-rats (*Heterocephalus glaber*; NMRs) are hystricomorphic rodents, which live in subterranean colonies in semi-arid regions of East Africa (Kenya, Somalia and Ethiopia). This species, characterized by many unique biological features, was first described in the mid-nineteenth century^1^, but in the last 3 decades, scientific interest has exponentially grown^2^. NMRs are one of the few known eusocial mammalian species, whose colony is dominated by a single reproductively active queen surrounded by usually 1–3 reproductive males^3^, although recent data suggest that only one dominant male is contributing to the progeny^4^. The rest of the animals, called workers, are sexually inactive^2–5^ and they have different tasks, such as taking care of offspring, defending the burrow system, and digging tunnels for foraging^5,6^. Subordinates and reproductively active animals differ in both behavior and anatomy^7^. In the case of female workers, reproduction is suppressed by social factors and can be resumed if the queen is removed^7,8^. Typical features of subordinate females are anovulation, prepubescent ovaries, small uterine horns, and not perforated vagina^9,10^. The socially induced block is the result of lower hormonal levels in the plasma of non-breeding females: in particular, low concentration of luteinizing hormone (LH) has been found, caused by low production of gonadotropin-releasing hormone (GnRH), there were also low levels of progesterone. Interestingly, no difference in plasma estradiol was observed^11^. The ovarian cycle of a NMR queen is approximately 34.4 days with 6 days of follicular phase and 27.5 days of a prolonged luteal phase^7^. Subordinate females can begin ovulating as early as 6 months old and they are capable of maintaining their fertility throughout their entire adult life^12^. Place and colleagues^13^ described unusually large ovarian reserve in 6-month-old females, ten times higher than other species of similar size. Moreover, they observed postnatal germ cell nests with proliferative potential, in both reproductively suppressed and active young females, which is a unique feature among mammals. This suggests that the process of oogenesis in NMRs’ ovaries occurs postnatally and plays a role in maintaining an exceptionally large ovarian reserve^14^.

Non-breeding males are characterized by smaller testicular size, persistent spermatogenesis with low sperm quality, and it has been found that plasma levels of testosterone are lower compared to breeders^11^.

Together with their extraordinary social organization and reproductive strategy, NMRs also have a very long lifespan, with a maximum-recorded age of 37 years^15^. Moreover, they appear to be cancer resistant^16,17^, even though Taylor and colleagues^18^ did report the presence of mainly benign neoplasia in some rare cases. Cellular mechanisms responsible for this feature are still unknown, but the presence of high-molecular hyaluronan in the extracellular matrix^19,20^, and their very stable epigenome^21^ may play a role. To fully understand the fundamental cellular mechanisms involved in all the above-mentioned characteristics, it is crucial to use pluripotent cell lines for in vitro studies. It has been shown that the transformation of somatic cells into induced pluripotent stem cells with the standard reprogramming factors such as OCT4, SOX2, KLF4, and c-Myc, was not efficient and the obtained cell lines had very low teratoma-forming tumorigenicity^21–24^. Another approach to obtaining pluripotent cells is the establishment of embryonic stem cells (ESCs) obtained directly from NMRs’ embryos. In different animal models, ESCs are derived from early blastocysts, which are either produced in vitro or flushed out from oviducts^25–28^. In the case of NMRs’ colonies, the sacrifice of the queen for embryo flushing would put the entire colony at risk because of the aggressive behavior for the establishment of a new hierarchy, and this will lead to many injuries and casualties over several months. To avoid this scenario, we decided to use the alternative approach: obtaining viable embryos through Assisted Reproduction Technologies (ARTs) from non-breeding females. The parameters of the NMRs’ sperm count restrict the ICSI as the only method for in vitro fertilization. The average concentration of the samples is 43 × 10^6^ spermatozoa/ml, but the average motility is very low (7.3%). Moreover, NMRs have very small polymorphic spermatozoa (average length 33.13 µm) with irregularly shaped heads, necks, and poorly defined midpieces and tails. The reason for such low sperm quality might be the lack of sperm competition among males due to their specific reproductive strategy^29^.

In this study, we present for the first time a protocol for in vitro production of NMRs’ embryos and blastocysts, starting from oocyte retrieval from female workers post-mortem, as a first step for future establishment of ESCs line.

## Results

The initial steps were the establishment of a suitable culture medium with optimal parameters for the in vitro maturation (IVM) of immature oocytes and the assessment of meiosis resumption (experiment I). Simultaneously to oocyte maturation, the expansion of the COCs happens, which is a marker that positively correlates with the quality of the IVM^30^. To follow these changes during IVM, we used a time-lapse incubator-system at 32 °C.

Matured oocytes were successfully fertilized using piezo-ICSI technique and presumptive zygotes were cultivated in vitro (experiment II). Because of unknown conditions of in vitro embryo culture (IVEC), two different approaches were used: the first one was the use of different culture media in conventional IVEC; in the second one, the zygotes were cultivated in isolated mouse oviducts in air–liquid interphase.

(Fig. 1).

**Figure 1:**
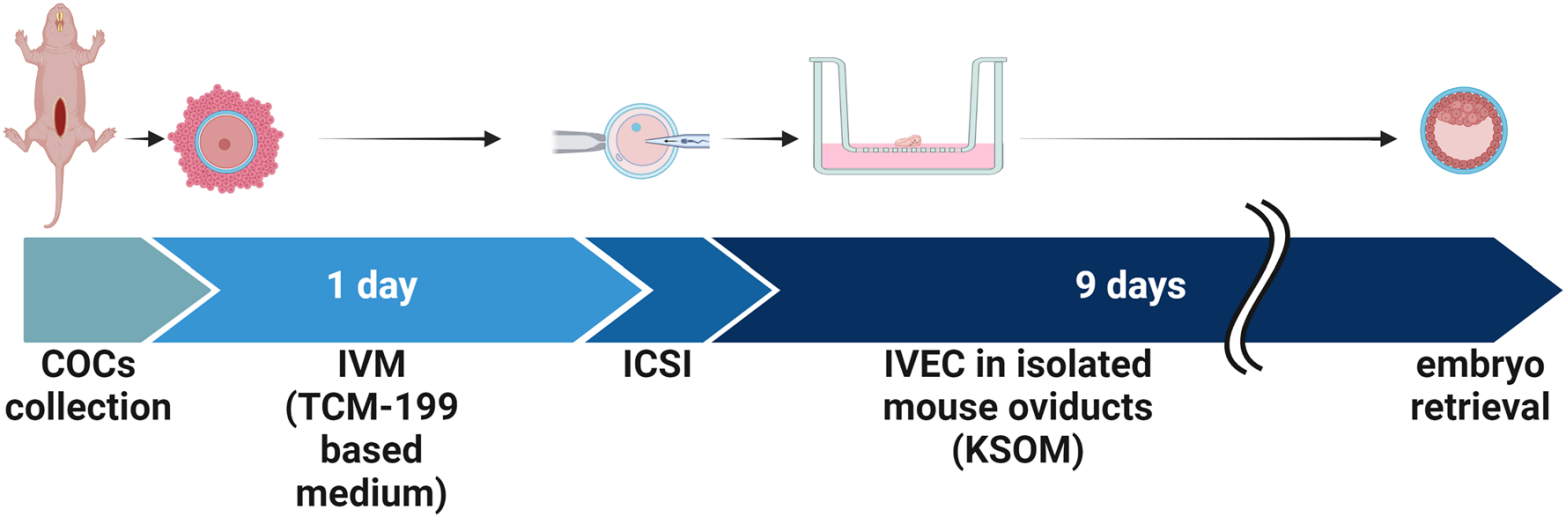
Schematic timeline of the in vitro production of NMRs' embryos using isolated mouse oviducts. Created with BioRender.com.

### Experiment I: Establishment of right IVM medium, determination of optimal IVM length, and assessment of cumulus expansion

#### Establishment of the IVM medium and culture condition

The highest percentage of MII oocytes was observed after 24 h of IVM. Therefore, in experiment II, 24 h of IVM was considered as standard.

#### NMR oocyte morphology

Pictures of denuded oocytes were analyzed via ImageJ for the assessment of the morphology and the measurements. 81 oocytes were measured with ImageJ and the average diameter was 101.41 µm ± 6.05 µm (average ± SD). The smallest had a diameter of 86.32 µm and the largest of 115.09 µm. The thickness of the zona pellucida was also measured and it was 9.61 µm ± 3.84 µm (average ± SD).

#### Assessment of kinetic of meiosis resumption

**Figure 2:**
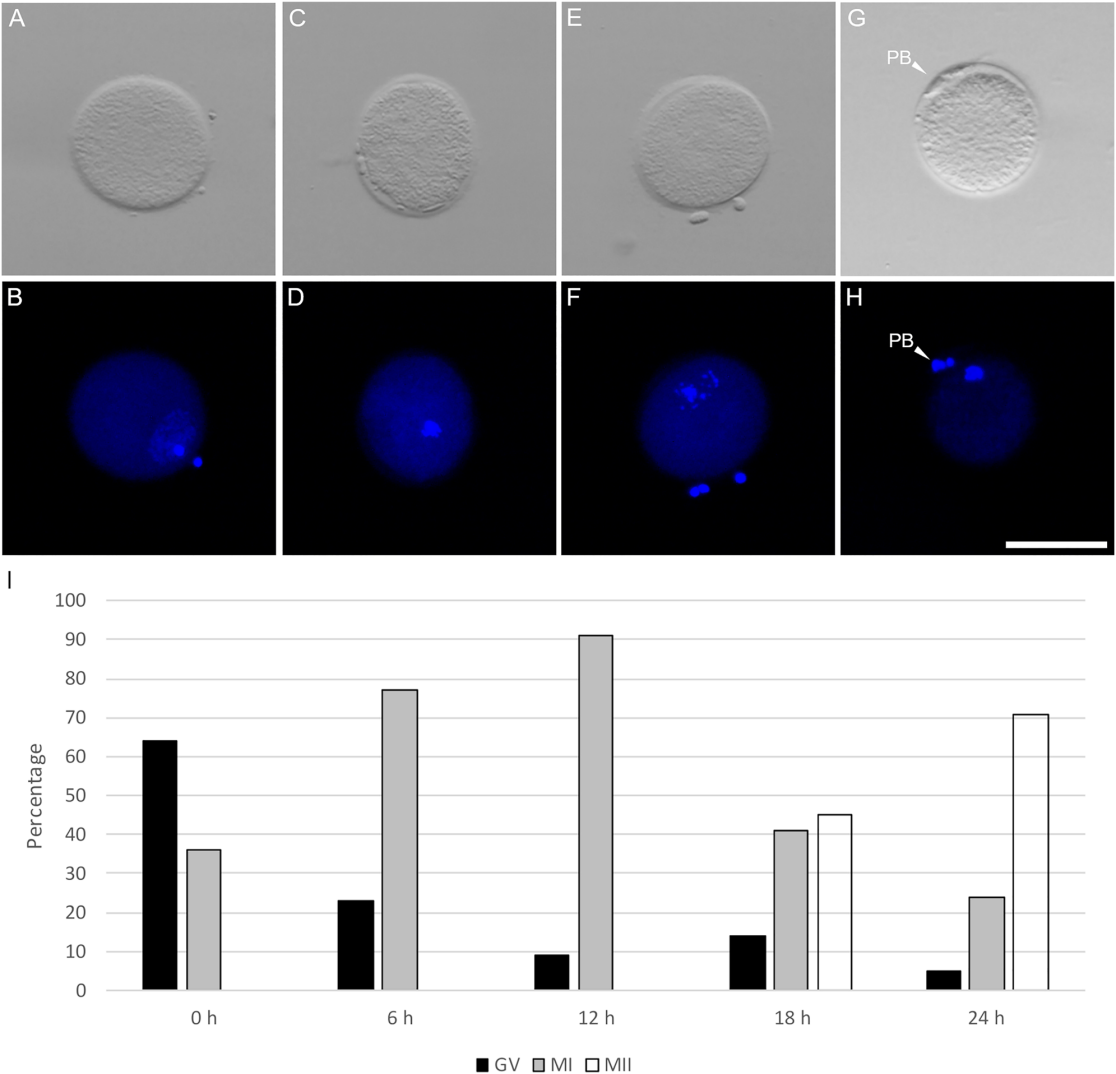
Nuclear status of developmental stages of NMR oocytes during IVM in bright and dark field. (**A**,**B**) GV stage; (**C-D)**: MI stage; (**E,F**) anaphase – telophase of MI; (**G**,**H**) MII stage. Scale bar = 100 µm; PB = polar body; (**I**) Representation of the developmental stages of NMR oocytes during IVM. At t = 0 h, 14 oocytes were classified as GV, and 8 were MI; At t = 6 h, 5 oocytes were GV and 17 were MI. At t = 12 h, 2 oocytes were GV and 20 were MI. At t = 18 h, 3 oocytes were GV, 9 were MI and 10 oocytes were classified as MII. At t =  ≥ 24 h, 1 oocyte was still in the GV stage, 10 were MI, and 34 were in the MII stage. GV = germinal vesicle; MI = metaphase I; MII = metaphase II.

133 oocytes were classified as a germinal vesicle (GV), metaphase I (MI), and MII according to the following parameters: (i) GV oocytes (Fig. 2A,B) are characterized by a large nucleus with decondensed chromatin, low brightness, and intact nuclear membrane; (ii) MI oocytes (Fig. 2C–F) were characterized broadly either as metaphase I (maximally condensed chromosomes, grape-like structure with a bright fluorescence) or telophase I/anaphase I transition (two distinct group of chromosomes in different stages of separation); (iii) MII oocytes (Fig. 2G,H) were clearly distinguished by condensed and tightly packed metaphase chromosomes, small and highly fluorescent PB. At the beginning of IVM, 63.64% of oocytes were in the GV stage with a visible nucleus. The proportion of MI oocytes rapidly increased, reaching the maximum percentage after 12 h of IVM (90.91%). After 18 h of IVM, there was an even distribution between MI and MII oocytes (respectively 40.91% vs. 45.45%), and a higher proportion of MII oocytes was reached after 24 h of IVM, with 71.43% matured oocytes (Fig. 2I). The prolonged culture up to 48 h did not change the proportion of matured oocytes.

#### Cumulus expansion analysis

**Figure 3:**
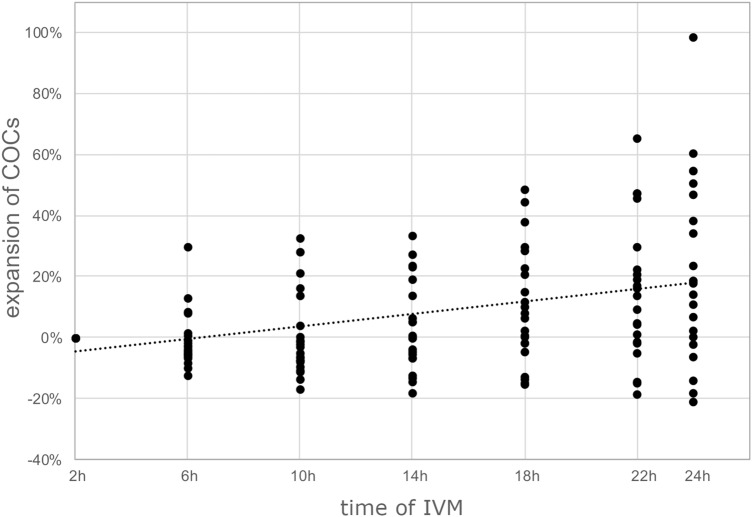
Analysis of cumulus expansion during IVM. Visual representation of the enlargement of the COCs during IVM. Each dot represents individual COC.

The surface of each cumulus-oocyte-complex (COC) was analyzed and compared to the original state at the beginning of the culture. COCs collected from follicles in sexually suppressed females manifest no relative enlargement of their surface. After 24 h of culture, there was an expansion of approximately 20% (Fig. 3). No statistical differences were found comparing subsequent time points (p > 0.05).

### Experiment II: fertilization of matured oocytes via piezo- ICSI and in vitro culture of NMRs’ embryos

#### Fertilization assessment

**Figure 4:**
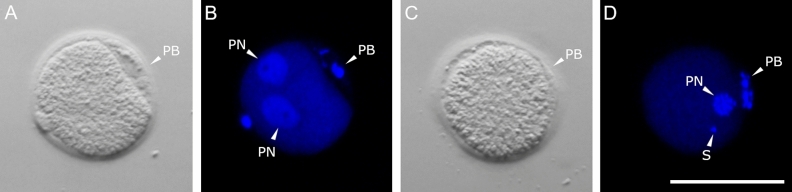
Fertility assessment of presumptive zygotes fixed 24 h after ICSI and stained with Hoechst 33258. (**A**,**B**) Fertilized oocyte with two visible uncondensed PN. (**C**,**D**) Unfertilized oocyte with maternal pronucleus and condensed sperm chromatin. PN = pronucleus, PB = polar body. Scale bar = 100 µm.

Injected oocytes were classified as fertilized when they showed 2 decondensed PN in the center of the ooplasm (Fig. 4A,B) and not fertilized when the condensed DNA from the sperm head was observed (Fig. 4C,D).

#### In vitro embryo culture

**Figure 5:**
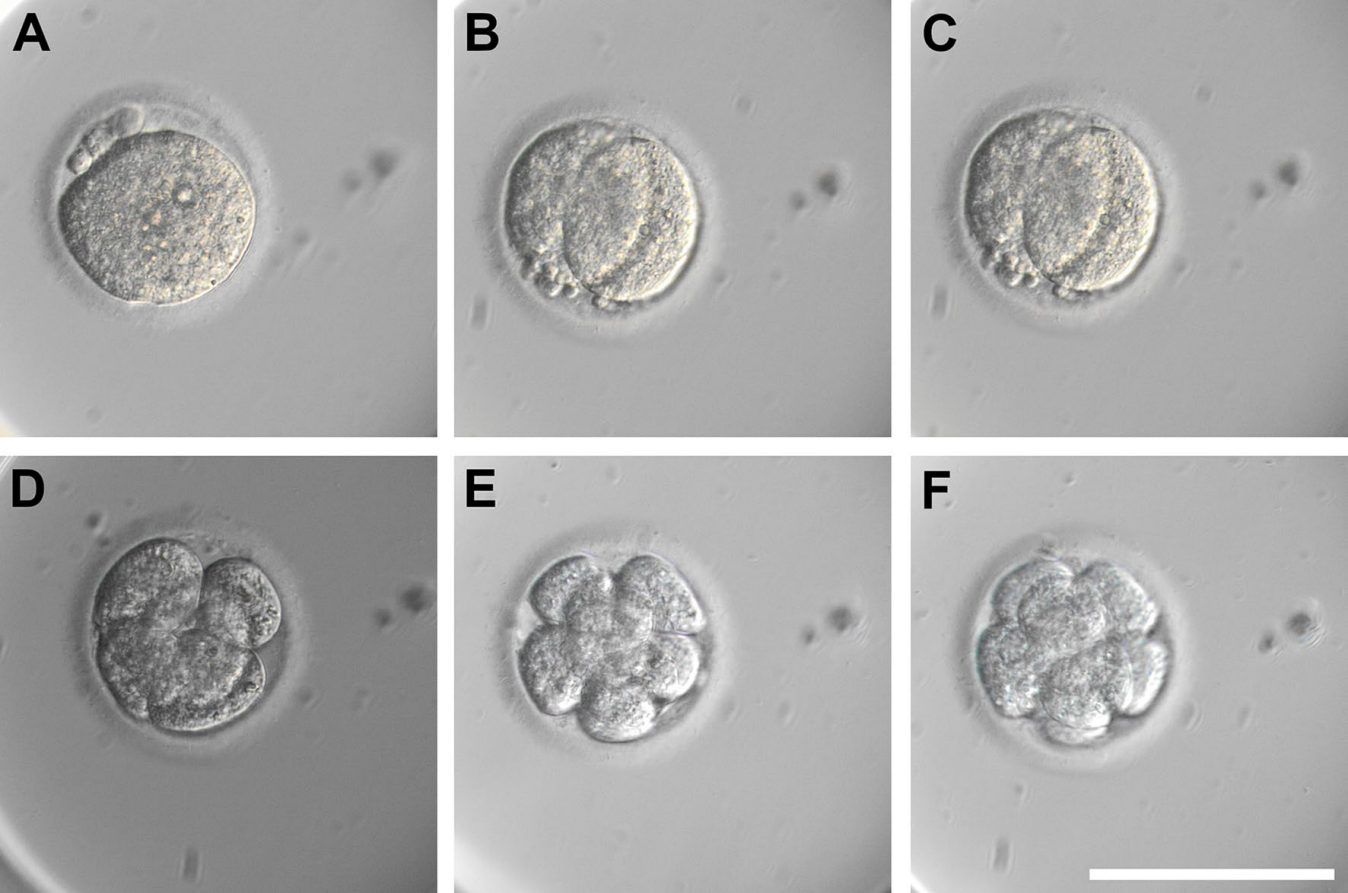
Cleavage-stage embryos. (**A**) PN formation 24 hpi; (**B**,**C**) 2-cell embryo 48 and 72 hpi; (**D**) 5-cells 96 hpi; (**E**) 6-cells embryo 120 hpi; (**F**) 8-cells embryo 144 hpi. Scale bar = 100 µm.

Embryos cultured in conventional IVEC reached the maximum number of cells (9) at day 6. A longer culture period did not lead to further development and no signs of compaction were observed (Fig. 5A–F). The first cleavage was observed 48 h post insemination (hpi). Therefore, based on this observation, the cleavage rate was calculated as the yield of cleaved embryos on the total number of zygotes in culture after 48 h. Cleavage rates in commercial KSOM and mSOF groups were respectively 42.65% and 50.00% (Table 1). No statistical difference was observed between the conditions.

**Table 1:** Comparison of the cleavage rate 48 hpi in commercial KSOM vs. mSOF culture media.

Culture medium	No. zygotes in culture	No. of cleaved embryos 48 hpi	Cleavage rate (%)
*KSOM*	167	58	42.03^a^
*mSOF*	44	17	50.00^a^

^a^No statistical differences were found in the embryo cleavage (p > 0.05).

**Figure 6:**
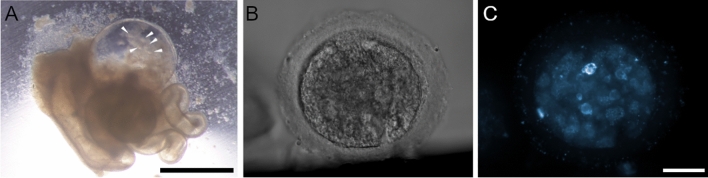
Embryo culture in isolated mouse oviducts and representative obtained blastocyst. (**A**) isolated mouse oviduct during embryo culture. Scale bar = 1 mm; (**B**,**C**) Obtained NMR blastocyst in phase contrast and fluorescence 3D image with nuclei stained with Hoechst 33258 (confocal microscopy). Scale bar = 20 µm.

On the contrary, from the group cultivated in isolated mouse oviducts (Fig. 6A), 5 early blastocysts were retrieved (Fig. 6B,C) after 9 days of embryo culture (22.73%) (Supplementary video 1). No viable embryos were retrieved from the oviducts flushed later.

## Discussion

Here we report the first successful in vitro production of NMRs’ blastocysts, derived from immature COCs retrieved from ovaries post mortem. After retrieval, COCs underwent the IVM first. During this process, cumulus cells surrounding the oocyte produce hyaluronic acid, which is secreted into the extracellular matrix. Despite the high maturation rate recorded (74.49%), we observed a very minimal cumulus size enlargement (~ 20%), if compared to other species, such as mice, pigs, and cattle^31–33^. This poor response of NMRs cumulus cells to hormonal stimulation could suggest some limitation of our IVM protocol, even though nuclear maturation was achieved and matured oocytes were successfully fertilized. The successful piezo-ICSI fertilization was proven by the presence of male and female PN. An alternative method to verify if ICSI is harmful to NMRs’ oocytes is to use the classic in vitro fertilization, i.e. the co-incubation of COCs using several hundred thousand spermatozoa. Unfortunately, this method is not applicable in this species due to the specific characteristics of the NMRs’ ejaculate: (i) low percentage of progressive motile spermatozoa; (ii) low count of sperm cells; (iii) low ejaculate volume (1–15 µl), and (iv) high percentage of teratospermia^29,34^.

Fertilized oocytes were cultured with two different approaches: (i) conventional IVEC system, and (ii) isolated murine oviducts in air–liquid interphase, which resulted in blastocyst formation after 9 days of culture. Surprisingly, the different temperature for in vitro culture, based on the respective body temperatures (32 °C in NMR vs. 37 °C in mouse) did not play a major role in the NMRs’ embryo development. It was proved that isolated mouse oviducts could support the development of mouse embryos to the blastocyst stage in vitro^35^. Moreover, mouse ampullas can even provide suitable conditions for further development of hamster 2-cell embryos^36^, and overcome the 2-cell block^37^. The species-unspecific nature of this type of culture was repeatedly proved by producing rat^38–40^, porcine^41^ and bovine blastocysts^42,43^. Contrarily, we were not able to find a proper strategy to support the activation of the embryo genome in the conventional IVEC. It is generally known that in vitro-produced embryos can exhibit developmental block at various stages of early development. In 1964, Yanagimachi and Chang^44^ observed in golden hamsters that in vitro-produced embryos could not be cultured further than two-cells stage. Schini and Bavister^37^ for the first time did prove that this developmental block was enhanced by the presence of phosphate and glucose in the culture medium and the employment of a free glucose and phosphate medium (HECM-1) helped to overcome this block and to support the development of a blastocyst.

In rat IVEC, the presence of glucose had no inhibitory effect on embryo development^45^. Further improvement of the HECM-1 medium with the addition of the 20 amino acids (mR1ECM) led to a rapid increase in the blastocyst formation rate^46^. In mouse IVEC, the presence of glucose in the standard M16 medium for the first 24 h of culture inhibited the embryo development, but this substrate is necessary for the progression to the blastocyst stage. This inhibitory effect was mitigated by the presence of pyruvate during the first 24 h of culture^47^.

Changes in the requirements of carbon source could also be the reason for a developmental block in NMRs’ embryos. Since NMRs belong to the rodent order, we tested the aforementioned media (HECM-1, mR1ECM, and M16 with different energy substrates) for NMR’s IVEC. The embryos did not overcome the 8-cell block, in addition to this, the percentage of degenerated embryos after 3 days of culture was higher compared to KSOM and mSOF media (Supplementary Table 1).

In our experiments, the oviducts remained active up to day 9 of the culture with clear peristaltic waves. Preliminary experiments with isolated NMRs’ oviducts from non-breeding females showed no sign of peristalsis. It is probable that the fallopian tube can provide nutritional support for the developing NMR’s embryos and the key components to overcome the developmental block. The oviduct fluid is a complex solution of blood plasma and components secreted by oviduct epithelial cells. Its content differs within oviduct parts and stages of the estrus cycle^48,49^. Gardner and Leese^50^ reported that in mice a small volume of oviduct fluid (~ 20 nl) was sufficient for the analysis of nutrient concentration. Based on this data, a culture medium was elaborated and it was successfully used for the culture of embryos with a similar blastocyst formation rate as the standard M16 medium. A similar approach could be applied to the NMR; however, it would be crucial to obtain fluid from a queen in a specific phase of the oestrus cycle. A similar problem arises with the co-culture of embryos with the monolayer of oviduct epithelial cells. This technique offers another possibility for the embryo culture, due to the presence of the secretory cells. For example, cells obtained from oviducts isolated from cows in the non-luteal phase can support bovine embryo development to the blastocyst stage^51^. However, oviduct epithelial cells cultured in standard conditions do not maintain the epithelial phenotype and in longer culture they change both morphology and function^52^. Culture in air–liquid interphase can provide a more natural environment for these types of cells^53^, but the necessity of sacrificing breeding females remains. In this article, we have demonstrated that isolated mouse oviducts can support the development of NMR’s embryos until the blastocyst stage.

Nevertheless, this approach has some limitations: it is impossible to monitor the embryo development inside the oviduct lumen and therefore keep track of the subsequent embryo divisions. Moreover, we have to take into account the source of COCs: since they are retrieved post-mortem from non-breeding females and these animals are characterized by different hormonal profile when compared to the queen^11^ it is possible that their developmental potential is suboptimal and this would affect the success rate of our protocols. Nevertheless, we demonstrate that it is possible to produce viable blastocysts starting with COCs retrieved from worker animals.

Another issue is that little is known about the embryo development in NMR queens, since they are the only ones who naturally carry on a pregnancy. It could be interesting to obtain blastocysts in vitro, starting from queen’s COCs, but this approach was not applicable in our case; as mentioned before, the removal of a queen will jeopardize the whole colony, leading to many casualties for the establishment of the new hierarchy within the colony itself. Isolating a non-breeder to induce cycling is time-consuming and ethically questionable; NMRs are highly social animals and isolation causes stress to them^54^.

An additional limitation of this study is the absence of good fluorescent signal from immunostaining for pluripotency factor OCT4 (Santa Cruz Biotech, sc-5279, data not presented). Notably Lee et al.^23,24^ had previously used this antibody to detect the expression of OCT4 in iPSC, our experimental outcome did not replicate this observed pattern. Unfortunately, it was not possible to perform additional experiments to elucidate this inconsistency due to limited access to the biological material.

Nevertheless, this is the first step for the in vitro production of NMRs’ embryos. The future goal is to produce a chemically defined medium to overcome the 8-cell block, which will reflect the specific NMR requirements for the embryonic development.

## Materials and methods

### Ethics statement

Twelve experimental NMR colonies are kept at the Leibniz Institute for Zoo and Wildlife Research (IZW), Alfred-Kowalke-Str. 17, 10315, Berlin, Germany and they are annually inspected by the Regional Office for Health and Social Affairs Berlin, Germany (Landesamt für Gesundheit und Soziales, Berlin, Germany). Breeding allowance (#ZH 156) and ethical approval (T 0073/15) were granted by the Ethics Committee of the aforementioned office. All experiments were carried out in a laboratory located in the same building at the IZW and experimental protocols were approved by the internal committee of IZW. The study and all methods were performed in accordance with the German Animal Welfare Act (Tierschutzgesetz) and ARRIVE guidelines (https://arriveguidelines.org).

### Media

Unless otherwise stated, all chemicals were purchased from Sigma-Aldrich (Missouri, USA). COCs and oocytes outside the incubator were handled in a commercial M2 medium.

### Experiment I: Establishment of right IVM medium, determination of optimal IVM length, and assessment of cumulus expansion

#### COCs collection, IVM, and assessment of the kinetic of meiosis resumption

In the context of euthanasia for organ collection for different in vitro studies, ovaries were opportunistically collected from 4 female workers between 1 and 4 years old (Supplementary Table 2). The animals were anesthetized with isoflurane and euthanized by cervical dislocation. Ovaries were collected into phosphate-buffered saline (PBS—Biowest, France) at room temperature (RT), brought to the laboratory, and processed immediately. COCs were isolated by slicing the follicles with injection needles under a stereomicroscope on a 32 °C heated plate, and collected into pre-warmed M2 medium. The IVM medium used in this study was based on Jiao et al.^55^ with some modification: TCM-199 (Life technologies, CA, USA) supplemented with 1% (v/v) FCS, 0.22 mM sodium pyruvate, 0.05 IU/ml porcine FSH (ProSpec-Tany Technogene Ltd., Israel), 0.05 IU/ml hCG, 10 ng/ml EGF, and 50 ng/ml gentamicin.

133 COCs were cultivated for up to 48 h in a dish with pre-equilibrated IVM medium covered with oil at 32 °C in a humidified atmosphere of 5% CO_2_ and 5% O_2_ in air. At predetermined intervals of 6 h, 22 to 24 COCs were randomly sampled, treated with 80 IU/ml hyaluronidase (in TCM-199 + 20 mM HEPES), mechanically denuded by gentle pipetting and fixed at 4 °C with 3.7% (v/v) formaldehyde solution in PBS. After 24 h of fixation, they were stained with 2.5 µg/ml Hoechst 33258 in 3:1 glycerol/PBS, and mounted on microscope slides covered with coverslips, sealed with nail polish, and kept at 4 °C in the dark until observation with an epifluorescence stereomicroscope (Nikon SMZ18). Matured oocytes (MII) were defined as oocytes with a visible polar body (PB) and 2 sets of chromatin, identified by 2 separate fluorescence signals.

#### NMR IVM and cumulus expansion analysis

For these sets of experiments, COCs were isolated from ovaries as described before and they were put individually into a 96-well dish with IVM medium (established in Exp. I) covered with heavy mineral oil (Stroebech media, Denmark) and cultured for 24 h in a humidified atmosphere of 5% CO_2_ at 32 °C. Previous experiments showed no difference in maturation rates between COCs that matured in normoxia vs. COCs that matured in reduced oxygen. Pictures of COCs were obtained at intervals of 4 h using the IncuCyte^®^ Live-cell analysis system (Sartorius AG, Germany). Subsequently, pictures were analyzed with ImageJ software to evaluate the percentage of expansion during IVM. The cumulus projected area was measured as the number of pixels, and the percentage of increased surface area was calculated with the following equation:

$${exp}_{x}= \frac{{A}_{x}- {A}_{0}}{{A}_{0}}*100$$

where ${A}_{x}$ is the area of COCs at different time points, and ${A}_{0}$ is the area in the first obtained picture.

Data were analyzed using the Student’s t-test among groups at different time points. The level of significance was set at p < 0.05.

### Experiment II: fertilization of matured oocytes via piezo-ICSI and in vitro culture of NMRs’ embryos

#### COCs collection and IVM

COCs were retrieved from 16 females (Supplementary Table 2) as described before, and they were put in IVM in a humidified atmosphere of 5% CO_2_ at 32 °C for 24 h. At the end of IVM, COCs were treated with 80 IU/ml hyaluronidase and mechanically denuded by gentle pipetting. Oocytes with visible PB were selected for fertilization via piezo-ICSI. Oocytes without a visible PB were fixed and stained as described before for the assessment of the nuclear status.

#### Semen collection and processing

Fresh semen was collected from different males opportunistically in the context of colony reproductive management via electroejaculation^34^. For each collection, the very low amount of ejaculate retrieved (1–15 µl) was diluted in 500 µl of pre-warmed HEPES-TALP medium^56^, and centrifuged at 700*g* for 10 min. The pellet was resuspended in 500 µl of pre-warmed HEPES-TALP medium, and a drop was put on a glass slide for the assessment of progressive motility and total number of spermatozoa.

#### Piezo-ICSI

ICSI was performed on an inverted microscope Olympus IX73 (Olympus, Japan) coupled with Transferman micromanipulators, Cell Tram 4e injectors and PiezoXpert (Eppendorf, Germany) at 32 °C on a heated plate.

A blunt needle filled with Fluorinert FC-770 with an inner diameter of 3 μm and an angle of 15° (Biomedical instruments, Germany) was used for injection. A holding needle with an inner diameter of 25 μm and an angle of 30˚ (Biomedical Instruments, Germany) was used for the holding of the oocytes.

The ICSI manipulation dish was prepared using one drop of HEPES-TALP, one drop of 10% (w/v) polyvinylpyrrolidone (PVP) in HEPES-TALP, and 3 drops of M2 medium under light mineral oil (Gynemed, Germany). A small amount of sperm suspension was transferred to the PVP drop, and a single spermatozoon was immobilized with multiple pulses applied on the tail region. For the penetration of zona pellucida, multiple pulses with intensity 10 and speed 8 were used. The elastic oolema was penetrated with a single pulse with intensity 8 and speed 5. A total of 368 oocytes were injected.

#### Embryo culture

After ICSI, presumptive zygotes were randomly allocated into embryo culture dishes with pre-equilibrated media covered with light mineral oil. Two different culture media were tested: (1) Commercial EmbryoMax KSOM medium supplemented with amino acids, and (2) Synthetic oviduct fluid (mSOF) based on Tervit et al.^57^ with minor modifications. 165 presumptive zygotes were cultivated in commercial EmbryoMax KSOM, and 44 presumptive zygotes were cultivated in mSOF. Embryo culture lasted up to 10 days at 32 °C in a humidified atmosphere of 5% CO_2_ and 5% O_2_ in air. Culture dishes were shortly removed from the incubator for morphological developmental assessment and obtaining pictures every 24 h.

#### Fertilization assessment

The assessment of zygote development was done after 24 h of in vitro embryo culture (IVEC). Due to the granular ooplasm, pronuclei (PN) were not always visible under optical or stereomicroscope, therefore, randomly 10 presumptive zygotes were fixed, stained with Hoechst 33258, mounted on a glass slide, and observed under an epifluorescence microscope as described before.

#### Embryo culture in isolated mouse oviducts

The day before fertilization, 4 female BL6J mice were mated with vasectomized males. 18 h later, females with vaginal plugs were sacrificed and isolated oviducts were transported to the laboratory in commercial M2 medium. The excessive connective tissue around oviducts was removed under the stereo microscope and the isolated organs were placed on a trans-well plate (3414 Costar, Corning) over 1.8 ml of pre-equilibrated commercial KSOM medium. Presumptive zygotes produced in vitro were transferred with a blunt glass pipette to the ampullae of the isolated murine oviducts. Plates were incubated at 37 °C in 5% CO_2_ in the air with maximum humidity for up to 12 days; the culture medium was refreshed every second day. On days 9, 11, and 12, the embryos were flushed out of the oviduct with a pre-warmed M2 medium.

#### Staining of embryos

After flushing, the obtained embryos were fixed at RT in 4% (w/v) paraformaldehyde solution in PBS. After 30 min of fixation, embryos were stained with Hoechst 33258, mounted on a glass slide, and observed under an epifluorescence or confocal microscope.

### Supplementary Information

Supplementary Table 1.

Supplementary Table 2.

Supplementary Video 1.

## Data Availability

All data generated or analyzed during this study were included in the manuscript and its supplementary information files.
