# Supplementary material for: In vitro production of naked mole-rats’ blastocysts from non-breeding females using in vitro maturation and intracytoplasmic sperm injection
Source: Sci Rep. 2023 Dec 15;13:22355. doi: 10.1038/s41598-023-49661-6 (PMC10724253; doi:10.1038/s41598-023-49661-6)
Supplement: Supplementary file 1 — Supplementary Table 1. [file 41598_2023_49661_MOESM1_ESM.pdf]

Supplementary table 1. Cleavage rate and developmental stages of NMR embryos in different IVEC media

Medium	No. of zygotes in culture	cleavage rate 48hpi	Embryo stage at the end of IVEC (168hpi)			
			not cleaved	2 blastomers	4-15 blastomers	Degenerated
KSOM	165	58 (42.03%)	12 (7.27%)	21 (12.73%)	26 (15.76%)	108 (65.45%)
mSOF	44	17 (50.00%)	6 (13.64%)	15 (34.09%)	4 (9.09%)	19 (41.18%)
mR1ECM	22	3 (15.79%)	2 (9.09%)	1 (4.55%)	2 (9.09%)	17 (77.27%)
HECM1	30	10 (41.67%)	0 (0.00%)	2 (6.67%)	1 (3.33%)	27 (90.00%)
M16	11	4 (36.36%)	0 (0.00%)	0 (0.00%)	0 (0.00%)	11 (100.00%)

Cleavage rate = yield of cleaved embryos on total number of vital zygotes in culture after 48 h post insemination

Supplementary video 1. 3D visualization of NMR blastocyst on day 9 of IVEC acquired via confocal microscopy. Nuclei are stained with Hoechst 33258.
