# Supplementary material for: In vitro production of naked mole-rats’ blastocysts from non-breeding females using in vitro maturation and intracytoplasmic sperm injection
Source: Sci Rep. 2023 Dec 15;13:22355. doi: 10.1038/s41598-023-49661-6 (PMC10724253; doi:10.1038/s41598-023-49661-6)
Supplement: Supplementary file 2 — Supplementary Table 2. [file 41598_2023_49661_MOESM2_ESM.pdf]

Supplementary table 2: Animals used for *in vitro* experiments

experiment	animal's age (years)	animal's weight (g)	COCs retrieved
I	1	17.2	53
	1	22.1	57
	1.6	19.9	43
	3.1	24.3	51
II	2.4	34.6	18
	2.7	25.8	38
	2.7	22.6	40
	2.7	27.5	40
	1.8	23.7	20
	1.8	20.6	23
	2.1	19.8	22
	2.5	35.0	50
	2.3	28.4	50
	2.7	32.5	55
	2.6	28.2	49
	adult (uknown age)	65.0	48
	3.7	34.0	46
	2.4	24.5	45
	2.4	24.7	41
	5.4	19.9	28
		average = 27.52	average = 40.85
